# An A- and B-Site Substitutional Study of SrFeO_3−δ_ Perovskites for Solar Thermochemical Air Separation

**DOI:** 10.3390/ma13225123

**Published:** 2020-11-13

**Authors:** Tyler P. Farr, Nhu Pailes Nguyen, H. Evan Bush, Andrea Ambrosini, Peter G. Loutzenhiser

**Affiliations:** 1 George W. Woodruff School of Mechanical Engineering, Georgia Institute of Technology, Atlanta, GA 30332, USA; tyler.farr@gatech.edu (T.P.F.); nhu_q.nguyen@gatech.edu (N.P.N.); 2 Concentrating Solar Technologies, Sandia National Laboratories, Albuquerque, NM 87185, USA; hebush@sandia.gov (H.E.B.); aambros@sandia.gov (A.A.)

**Keywords:** SrFeO_3−δ_, air separation, concentrated solar

## Abstract

An A‑ and B‑site substitutional study of SrFeO_3−δ_ perovskites (A’*_x_*A_1−*x*_B’*_y_*B_1−*y*_O_3−δ_, where A = Sr and B = Fe) was performed for a two‑step solar thermochemical air separation cycle. The cycle steps encompass (1) the thermal reduction of A’*_x_*Sr_1−*x*_B’*_y_*Fe_1−*y*_O_3−δ_ driven by concentrated solar irradiation and (2) the oxidation of A’*_x_*Sr_1−*x*_B’*_y_*Fe_1−*y*_O_3−δ_ in air to remove O_2_, leaving N_2_. The oxidized A’*_x_*Sr_1−*x*_B’*_y_*Fe_1−*y*_O_3−δ_ is recycled back to the first step to complete the cycle, resulting in the separation of N_2_ from air and concentrated solar irradiation. A-site substitution fractions between 0 ≤ *x* ≤ 0.2 were examined for A’ = Ba, Ca, and La. B-site substitution fractions between 0 ≤ *y* ≤ 0.2 were examined for B’ = Cr, Cu, Co, and Mn. Samples were prepared with a modified Pechini method and characterized with X-ray diffractometry. The mass changes and deviations from stoichiometry were evaluated with thermogravimetry in three screenings with temperature- and O_2_ pressure-swings between 573 and 1473 K and 20% O_2_/Ar and 100% Ar at 1 bar, respectively. A’ = Ba or La and B’ = Co resulted in the most improved redox capacities amongst temperature- and O_2_ pressure-swing experiments.

## 1. Introduction

N_2_ is an industrial gas with a wide array of chemical and medical applications, including in the production of ammonia via the Haber–Bosch process [1]. Current practice to obtain N_2_ employs cryogenic air separation to compress and liquefy the air followed by distillation to separate O_2_ and N_2_. The best cryogenic separation processes operate with energy demands three times higher than the thermodynamic minimum energy required for N_2_/O_2_ separation [2]. Pressure-swing adsorption is another air separation process utilizing activated carbon, but with the limitation of not producing high-purity N_2_ [3,4]. Inorganic membranes provide energy-efficient and scalable means of gas separation but are often tailored towards CO_2_, with little effectiveness for air separation [5]. Chemical looping air separation relies on reversible reduction/oxidation (redox) reactions to cyclically adsorb O_2_ from the air to produce high-purity N_2_ [6].

**Figure 1:**
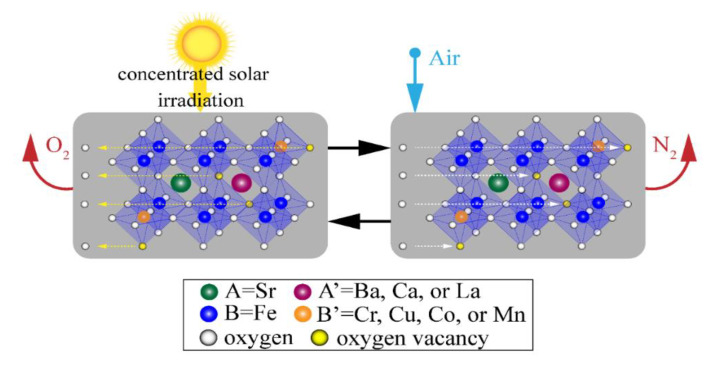
Schematic of the two-step solar thermochemical air separation cycle based on reversible reduction/oxidation SrFeO_3−δ_ perovskite reactions with different A- and/or B-site substitutions.

SrFeO_3−δ_ (δ ≡ deviation from stoichiometry) is a redox-active mixed ionic-electronic conducting material that is particularly attractive for reversible redox cycling. High redox capacities in the absence of crystal structure changes and thermal stability over a large range of temperature- and O_2_ partial pressure make SrFeO_3−δ_ ideal for solar thermochemical air separation [7,8]. Rapid kinetics result, in part, from the facile conduction of electrons and O^2−^ ions through the sublattice at elevated temperatures. This phenomenon produces charge imbalances which create O^2−^ vacancies in the sublattice. The two-step solar thermochemical cycle to produce N_2_ from air operates via the reversible SrFeO_3−δ_ redox reaction, represented in Kröger–Vink notation as:

(1) $${2\mathrm{Fe}}_{\mathrm{Fe}}^{x}{+O}_{O}^{x}⇌{2\mathrm{Fe}}_{\mathrm{Fe}}^{\prime }{+V}_{ö}+\frac{1}{2}O_{2}\left(g\right)$$

where V_Ö_ represents an oxygen vacancy; ${\mathrm{Fe}}_{\mathrm{Fe}}^{\times }$ and $O_{O}^{\times }$ are the neutral charged ions; ${\mathrm{Fe}}_{\mathrm{Fe}}^{\prime }$ is the negatively charged ion; and the non-labile Sr^2+^ is omitted. The two-step solar thermochemical cycle for air separation depicted in Figure 1 encompasses the following steps:

- The thermal reduction of Fe^4+^ → Fe^3+^ on the B-site using concentrated solar irradiation as process heat at elevated temperatures and low O_2_ partial pressures, where V_Ö_ are formed as 2O^2−^ → O_2_(g) to maintain electroneutrality.
- The oxidation in air at lower temperatures and elevated O_2_ partial pressure to produce N_2_ as Fe^3+^ → Fe^4+^, V_Ö_ sites are filled, and the SrFeO_3−δ_ is recycled back to the first step to complete the cycle.

The net result of the cycle is that O_2_ is removed from the air with concentrated solar irradiation to produce N_2_. A- and B-site cation substitutions are useful levers to tune the thermodynamic properties of SrFeO_3−δ_. Important thermodynamic properties for solar thermochemical air separation are low thermal reduction temperature, high redox capacity, and low reaction enthalpy. A low average reaction enthalpy of 166 ± 5 kJ per mol O_2_ from δ = 0 to 0.5 was determined for SrFeO_3−δ_ [9], which corresponds to a lower energy input per mol of O_2_ captured compared to CaMnO_3−δ_ [10]. This results in a decrease in the concentrated solar irradiation necessary to drive the cycle. A comprehensive understanding of the thermodynamics and rate limiting mechanism(s) is foundational for designing solar thermochemical reactors coupled to solar concentrating facilities. Thermodynamic and kinetic characterizations are necessary to optimize absorption efficiencies coupled to operating temperatures, reaction extents, and residence times in a reactor design [11,12].

Examination of A- and B-site substitution in SrFeO_3−δ_ (A’*_x_*A_1−*x*_B’*_y_*B_1−*y*_O_3−δ_ where A = Sr, for A-site substitution fractions of 0 ≤ *x* ≤ 0.2, and B = Fe for B-site substitution fractions of 0 ≤ *y* ≤ 0.2) is necessary to further refine performance aimed at increasing redox capacity and promoting long term cyclability, structural stability, and enhanced kinetics. Isovalent A-site substitutions with alkaline earth metals have been shown to improve oxygen vacancy formation [13] and varying the A-site atomic radii shows a strong trend with O^2−^ ion conductivity [14]. Aliovalent A-site substitutions (e.g., La^3+^) have been explored as a mechanism to alter the thermodynamic properties by changing the average site charge on the B-site [15]. Previous studies conducted with La*_x_*Sr_1−*x*_Co*_y_*[Mn, Fe]_1−*y*_O_3−δ_ and Ba*_x_*Sr_1−*x*_CoO_3−δ_ have demonstrated the influence of crystal structure and substituent concentration on redox performance [16,17]. B-site substitution with Co, Cu, and other metal cations are potential levers towards increased reducibility while maintaining the crystallographic stability of SrFeO_3−δ_ [8]. B’ = Al compounds have been investigated, resulting in a decrease in the deviation from stoichiometry and in the reduction extent [9]. A’ = Ca and B’ = Co compounds have been investigated and showed improved oxidation kinetics with comparable redox capacities to that of SrFeO_3−δ_ [18,19]. Other studies of B’ = Co and Cu have been conducted due to the relatively low reaction enthalpies; however, they were not thermodynamically stable [8,20].

In this work, A’*_x_*Sr_1−*x*_B’*_y_*Fe_1−*y*_O_3−δ_ was investigated for solar thermochemical air separation cycles where A’ = La, Ba, and Ca for A-site fractions of 0 ≤ *x* ≤ 0.2; and B’ = Co, Cr, Cu, and Mn for B-site fractions of 0 ≤ *y* ≤ 0.2. Samples were synthesized using a modified sol-gel method and characterized with X-ray diffractometry and scanning electron microscopy with energy dispersive spectroscopy. Temperature- and O_2_ pressure-swing thermogravimetry was performed to analyze the redox capacity and identify promising candidates for solar thermochemical air separation applications. The schematic for a two-step solar thermochemical air separation cycle is depicted in Figure 1.

## 2. Materials and Methods

Synthesis Methodology: Samples were synthesized using a modified Pechini method [21] from metal nitrate salt precursors (ALFA AESAR, ≥98% purity), dissolved in stoichiometric ratios in ultrapure H_2_O, with citric acid as the chelating agent. The solutions were continuously heated and stirred on a hotplate to just below 373 K to promote evaporation. The stirring was halted at the onset of gel formation. The samples were dehydrated for 16 h in a drying oven at 373 K, broken up into powders, and heated on a hotplate to above 523 K to induce auto-ignition. The resulting powder was ground with an agate mortar and pestle and placed in alumina crucibles for calcination in a high‑temperature box furnace (Muffle Box Furnace, 4 × 4 × 5 in^3^, SentroTech, Strongville, OH, USA). The samples were first heated to 1073 K for 5 h to remove any remaining organic or nitrate compounds, re-ground, then subsequently heated to between 1473 and 1573 K for 24 to 48 h to produce the perovskite oxides.

Characterization: The crystal structure of each sample was determined via X-ray diffractometry (XRD, PANalytical X’Pert PRO Alpha-1 diffractometer with Cu Kα radiation, PANalytical, Malvern, UK) and compared against entries in the PDF-4+ Database [22]. Secondary phases were found to be <5% by mass when present. Particle morphology and elemental distribution were analyzed with scanning electron microscopy (SEM, Zeiss Ultra60 FE, Oberkochen, Germany) and energy-dispersive X-ray spectroscopy (EDS/EDX, Zeiss Ultra60 FE, Oberkochen, Germany). Scanning voltages of 8 to10 kV were utilized due to the low sample conductivity.

Thermogravimetry: Thermogravimetry (TGA, Netzsch STA 449 F3 Jupiter ±1 µg with three integrated mass flow controllers with precisions of ±1 mL_N_∙min^−1^ and accuracies of less than ±2% for N_2_, Selb, Germany) was used to examine redox capacities as a function of temperature- and O_2_ partial pressure. Powder samples were placed on an Al_2_O_3_ crucible shielded with a platinum foil (Sigma Aldrich 0.025MM Thick 99% Pt Foil 267244-1.4G, St. Louis, MO, USA) to prevent unwanted reactions with the sample holder, and sample temperatures were measured by an S-type thermocouple (±1.5 K) directly in contact with the crucible. The initial and final sample masses were measured with an analytical balance (Mettler Toledo ML54, ±0.1 mg, Columbus, OH, USA) to verify the total mass change measured with the TGA. A blank run with an empty crucible was run under identical conditions for each experiment to correct for the effects of buoyancy and gas dynamics.

Three suites of TGA screenings were run under different conditions to assess the viability of different A’*_x_*Sr_1−*x*_B’*_y_*Fe_1−*y*_O_3−δ_: (1) temperature-swing cycles to compare redox capacities; (2) temperature- and O_2_ pressure-swing cycles to examine redox capacities over multiple cycles for promising materials from TGA Screening 1; and (3) temperature- and O_2_ pressure-swings with additional cycles to examine structural stability and redox capacities for promising materials from the second TGA screening.

**Figure 2:**
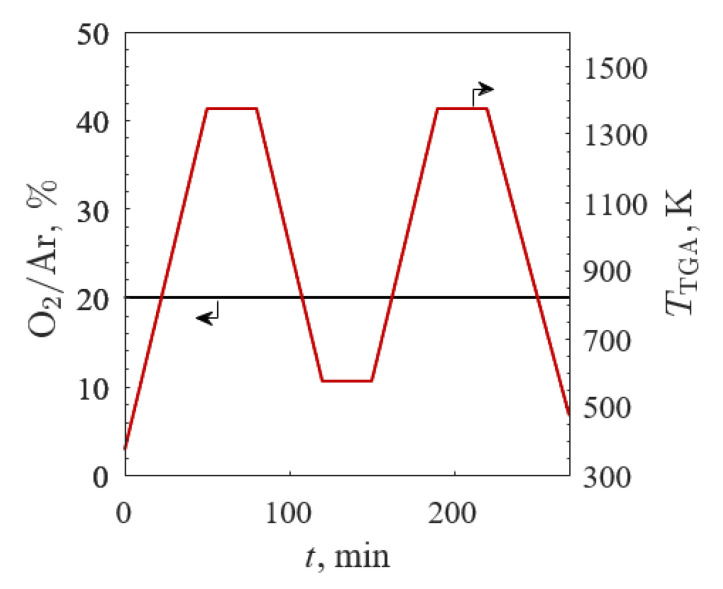
O_2_ concentration in Ar (black line) and temperature (red line) profiles versus time between 573 and 1373 K in 20% O_2_/Ar for the first TGA screening.

The first TGA screening examined all samples by varying the temperature between 573 K ≤ *T*_TGA_ ≤ 1373 K in 20% O_2_/Ar for two cycles, as depicted in Figure 2 with the O_2_/Ar (black line) and *T*_TGA_ (red line) profiles. TGA was run in 20% O_2_/Ar with a total gas flow of 100 mL_N_∙min^−1^ (where L_N_ refers to liters at standard conditions: 273 K and 1 bar) for all experiments. The samples were heated to 1373 K at 20 K/min and held isothermally for 30 min to allow the sample masses to equilibrate. The samples were then cooled to 573 K at 20 K/min and held isothermally for 30 min. This cycle was repeated to produce two identical stages, the first serving as the break-in cycle to off-gas unwanted adsorbed species and normalize the starting deviation from stoichiometry.

**Figure 3:**
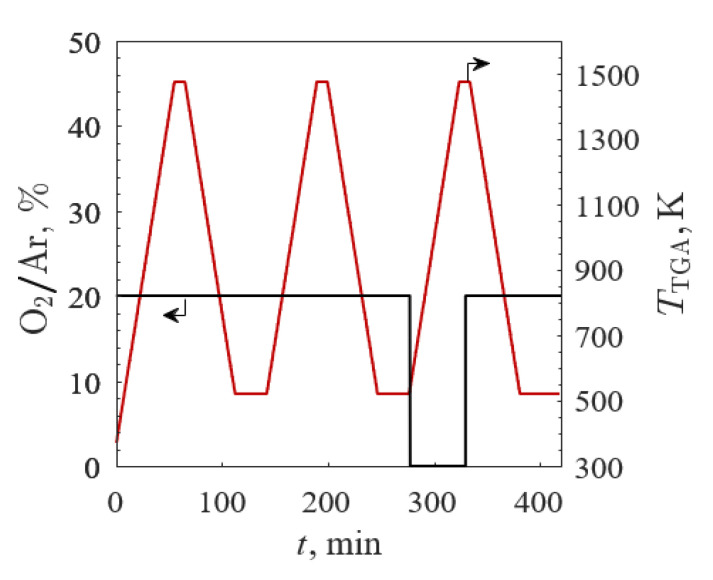
O_2_ concentration in Ar (black line) and temperature (red line) profiles versus time between 523 and 1473 K in 20% O_2_/Ar and 100% Ar for second TGA screening.

The second TGA screening was conducted for A’ = Ba, Ca, and La and B’ = Co, with O_2_/Ar (black line) and *T*_TGA_ (red line) profiles shown in Figure 3. The samples underwent three temperature-swing cycles between 523 and 1473 K. The first stage was a break-in cycle in 20% O_2_/Ar with a total gas flow of 100 mL_N_∙min^−1^ and a 20 K/min ramp rate. This was followed by an identical 20% O_2_/Ar cycle and a third cycle in 100% Ar. The 100% Ar reduction step was implemented to increase the reduction extent via O_2_ pressure-swing according to Le Chatelier’s Principle. The 100% Ar environment at 1473 K was maintained for 5 min before 20% O_2_/Ar was reintroduced with a total gas flow of 100 mL_N_∙min^−1^ and then cooled down to the ambient temperature at 20 K/min.

**Figure 4:**
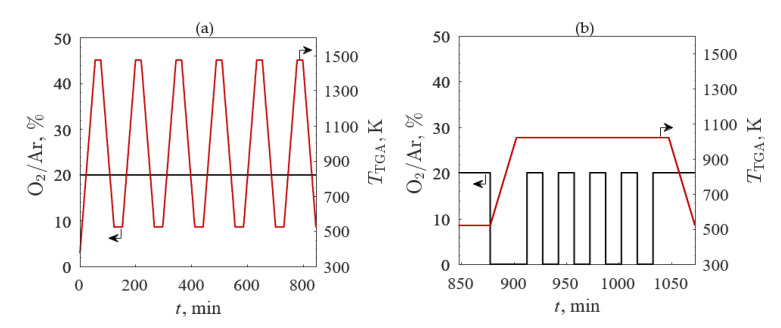
O_2_ concentration in Ar (black line) and temperature (red line) profiles versus time with the (**a**) temperature-swing between 523 and 1473 K in 20% O_2_/Ar and (**b**) O_2_ pressure-swing between 20% O_2_/Ar and 100% Ar at 1023 K for the third TGA screening.

The third TGA screening evaluated redox repeatability for the four samples with the highest redox capacities: (1) A’ = Ba, (2) A’ = Ba and B’ = Co, (3) A’ = La, and (4) A’ = La and B’ = Co. The O_2_/Ar (black line) and *T*_TGA_ (red line) profiles are shown in Figure 4 for (a) temperature- and (b) O_2_ pressure-swing cycles. TGA temperature- and O_2_ pressure-swings were performed to evaluate the redox capacity of each sample. After the initial break-in cycle, the samples were cycled between 523 and 1473 K in 20% O_2_/Ar five times with heating and cooling rates of 20 K/min and held isothermally for 20 min between heating and cooling steps. Five O_2_ pressure-swing cycles at an isotherm of 1023 K were consecutively completed for 20% O_2_/Ar and 100% Ar with the gas flow changed every 15 min. The samples were then cooled in 20% O_2_/Ar to ambient temperature at 20 K/min.

## 3. Results

A’*_x_*Sr_1−*x*_B’*_y_*Fe_1−*y*_O_3−δ_ with 0 ≤ *x,y* ≤ 0.2 was synthesized. Synthesis was limited to *x* and *y* ≤ 0.2 to study incremental changes in the SrFeO_3_ structure. Higher x and y did not always result in single phase perovskite oxides and were not examined further. Ba^2+^ and Ca^2+^ were selected as A-site substituents based on similar ionic radii to Sr^2+^ (ionic radii of 135, 100, and 118 pm, respectively [23]). La^3+^ (ionic radii of 103 pm [23]) was selected as an A-site substituent to examine the impact of increasing average A-site charge while decreasing average B-site charge. First-row transition metals with multiple oxidation states (e.g., Co, Cr, Cu, and Mn) were selected for B-site substitution due to strong favorability for forming stable perovskite oxides as predicted from density functional theory modeling [24]. The standard first letter convention for perovskite oxides, followed by the site fractions (e.g., Ba_0.2_Sr_0.8_FeO_3−δ_ ≡ BSF280 and SrCo_0.1_Fe_0.9_O_3−δ_ ≡ SCF019), was used in this work to identify different A’*_x_*Sr_1−*x*_B’*_y_*Fe_1−*y*_O_3−δ_. The convention was adjusted to include the full elemental symbols for Ca, Cr, and Cu with C representing Co.

### 3.1. Characterization

**Figure 5:**
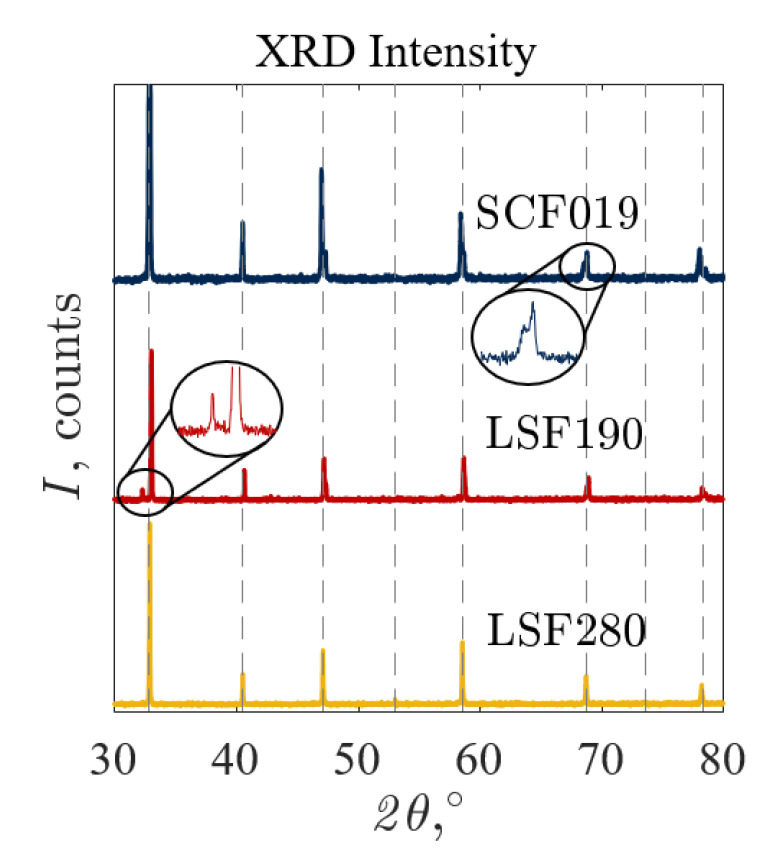
Intensity as function of 2θ from X-ray diffraction where the vertical dashed lines represent cubic SrFeO_3−δ_ (PDF 04 007 9408) compared with SrCo_0.1_Fe_0.9_O_3−δ_ (top blue), La_0.1_Sr_0.9_FeO_3−δ_ (middle red), and La_0.2_Sr_0.8_FeO_3−δ_ (bottom yellow).

XRD was used to determine the crystal structures of A’*_x_*Sr_1−*x*_B’*_y_*Fe_1−*y*_O_3−δ_. The diffractograms are shown in Figure 5 for SCoF019 (top blue), LSF190 (middle red), and LSF280 (bottom yellow) compared with SrFeO_3−δ_ (vertical dashed lines). Single-phase tetragonal or cubic perovskites with peaks similar to cubic SrFeO_3−δ_ were observed, except for LSF190, which shows a peak at 2θ = 32.3° that presumably corresponds to Sr_3_Fe_2_O_7−δ_. Peak splitting at 2θ = 69.6° for SCoF019 is indicative of a distortion from the cubic structure. An additional peak before the highest-intensity peak at 2θ = 32.3° for LSF190 indicative of a small amount of secondary phase. Some samples showed slight peak shifting from the cubic SrFeO_3−δ_ peaks, as seen in LSF280. This was due to an increase (shift to lower 2θ) or decrease (shift to higher 2θ) of the lattice parameters [15].

A summary of the identified crystal structures for all of the A’*_x_*Sr_1−*x*_B’*_y_*Fe_1−*y*_O_3−δ_ compositions is provided in Table 1. Melting was observed for B’ = Cu samples during synthesis with some samples such as SCuF280 experiencing incongruent melting during screenings, resulting in secondary phases of CuO and Fe_2_O_3_. Peak splitting in several of the patterns was indicative of lattice distortion. Peak splitting at 2θ = 69.6° as detailed in Figure 5 for SCoF019 was indicative of an orthorhombic structure.

**Figure 6:**
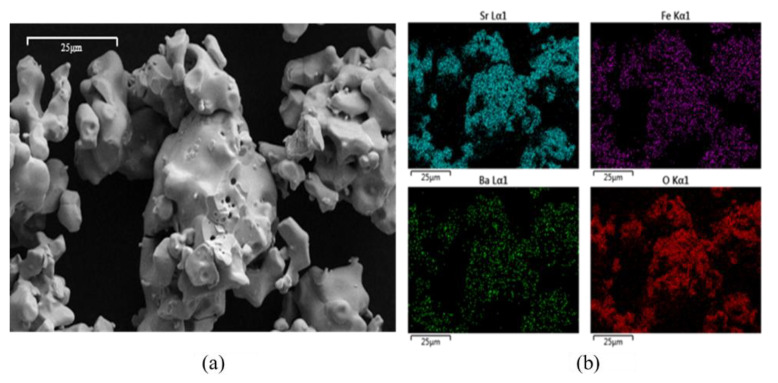
Images of Ba_0.1_Sr_0.9_FeO_3−δ_ from (**a**) scanning electron microscopy and (**b**) atomic distribution of Sr (blue), Fe (purple), and Ba (green) cations and O (red) anions from energy dispersive X-ray spectroscopy.

The particle morphology and elemental distribution for BSF190 were examined with SEM and EDS, shown in Figure 6 for (a) an SEM scan and (b) atomic distributions of Sr (blue), Fe (purple), Ba (green), and O (red) from EDS. SEM scans showed some particle rounding after cycling experiments with no evident changes in the particle size, suggesting no significant sintering. EDS maps showed uniform elemental distributions before and after the cycles. SEM and EDS showed similar effects for LSF190.

### 3.2. Thermogravimetry

The relative mass change and the deviation from stoichiometry change were used as metrics for determining redox capacity. The deviation from stoichiometry change is defined as:

(2) $$\Delta \delta \;=\;-2\frac{\Delta m}{m_{\mathrm{initial}}}\frac{M_{\mathrm{initial}}}{M_{O_{2}}}$$

where Δ*m* is the mass change relative to the initial mass corrected with the blank run; *m*_initial_ is the initial sample mass; $M_{O_{2}}$ is the molar mass of O_2_; and *M*_initial_ is the initial sample molar mass assuming stoichiometric oxygen in the sublattice.

#### 3.2.1. TGA Screening 1

**Figure 7:**
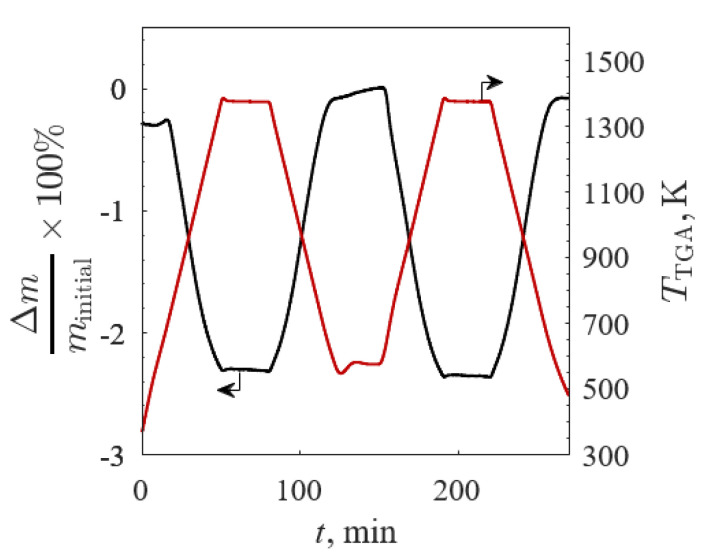
Relative mass change (black line) and temperature (red line) versus time of Ba_0.2_Sr_0.8_FeO_3−δ_ between 573 and 1373 K in 20% O_2_/Ar for TGA Screening 1.

The Δ*m*/*m*_initial_ (black line) and *T*_TGA_ (red line) versus time for BSCF190 for TGA Screening 1 are shown in Figure 7 (*m*_initial_ = 108.8 mg to ensure no mass transfer limitations). Decreasing and increasing Δ*m*/*m*_initial_ were directly correlated to increasing and decreasing *T*_TGA_, respectively, with virtually no lag during the changes from isothermal to non-isothermal steps, suggesting a heat transfer limited reaction. The differences between Δ*m*/*m*_initial_ for the successive cycles were small, indicative of little to no degradation in redox capacity between cycles.

A summary of the results for all the samples is given in Table 2 for TGA Screening 1 in terms of Δ*m*/*m*_initial_ and Δδ. Continuous redox behavior was observed for most samples as a function of *T*_TGA_. SCuF028 was the exception with sudden mass changes around 1473 K, presumably due to redox reactions of the secondary CuO/Cu_2_O phases. A-site substitution generally increased the redox capacities of every B-site substituted compound, most notably for A’ = Ba and La. The decreased redox capacity between LSF190 and LSF280 was in agreement with previously published work on LSF [25]. B-site substitution only increased the redox capacity for B’ = Co, and the redox capacities for B’ = Cr, Cu, and Mn were significantly reduced. The B’ = Mn and Cu redox capacities were consistent with previously published literature [26,27]. The results showed no significant dependence with *x* or *y* on the redox capacity except for B’ = Cr, which reduced the overall Δ*m*/*m*_initial_ from 1.78% (Δδ = 0.21) to 1.37% (Δδ = 0.16) as y increased from 0.10 to 0.20. B’ = Cr, Cu, and Mn were eliminated from subsequent TGA screenings due to their low redox capacities compared to SrFeO_3−δ_.

#### 3.2.2. TGA Screening 2

**Figure 8:**
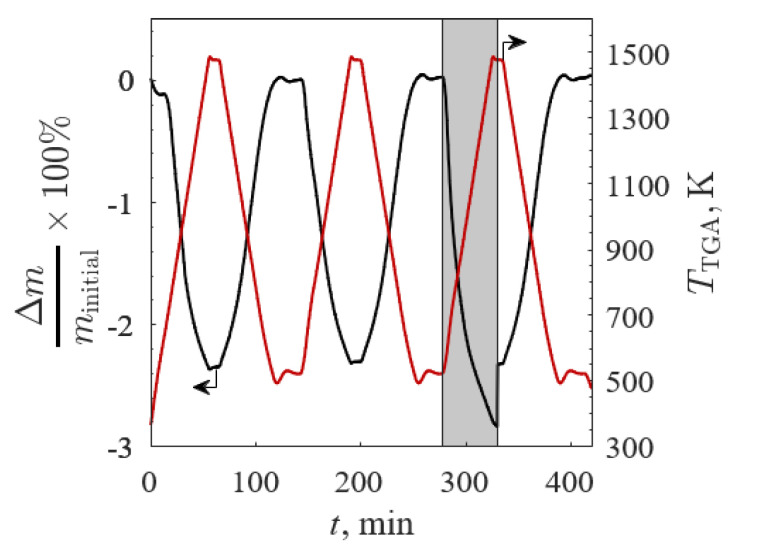
Relative mass change (black line) and temperature (red line) versus time of Ba_0.2_Sr_0.8_Co_0.2_Fe_0.8_O_3−δ_ between 523 and 1473 K in 20% O_2_/Ar and 100% Ar (gray region) for TGA Screening 2.

The Δ*m*/*m*_initial_ (black line) and *T*_TGA_ (red line) versus time for BSCF2828 for TGA Screening 2 are shown in Figure 8 (*m*_initial_ = 117.3 mg to ensure no mass transfer limitations), where 100% Ar is denoted by the gray shaded region. The profiles for Δ*m*/*m*_initial_ and Δδ were similar for each sample reoxidation as Δ*m* → 0 and Δδ → 0 in each of the three low temperature steps. The drop in Δ*m*/*m*_initial_ was caused by the gas switch during the isothermal stage from 100% Ar to 20% O_2_/Ar with a shift in chemical equilibrium according to Le Chatelier’s principle. The immediate response of Δ*m*/*m*_initial_ to the changing gas atmosphere was evidence of rapid oxidation. The Δ*m*/*m*_initial_ did not equilibrate during the allotted time for thermal reduction after switching to 100% Ar due to residual O_2_ during the gas changeover. The Δ*m*/*m*_initial_ for all samples closely followed the temperature and O_2_ pressure changes and quickly equilibrated under 20% O_2_/Ar, indicative of rapid reduction and oxidation reactions.

A summary of the results from TGA Screening 2 is given in Table 3 for Δ*m*/*m*_initial_ and Δδ. While B’ = Co improved the redox capacity in TGA Screening 1, this result was inconsistent with further analyses of analogs at higher *T*_TGA_ in both 100% Ar and 20% O_2_/Ar in the TGA Screening 2. All A-site substitutions improved the redox capacity in 20% O_2_/Ar with equal or improved results in 100% Ar. A’ = Ba and La had the greatest improvements in redox capacity. In all samples, *x* = 0.10 corresponded to a higher redox capacity compared to *x* = 0.20 except for CaSF190, which showed an increased redox capacity with increased x. Previous work has shown a positive trend for x for A’ = La and B’ = Co with sample redox capacity when reducing in 100% Ar from 473 to 1523 K [16]. While most samples did not equilibrate during the thermal reduction in 100% Ar, all A’ = Ca samples equilibrated faster for greater *x*. LSF and BSF190 (highlighted in Table 3) were the highest reducing samples.

A departure from the continuous reduction (and not oxidation) with *T*_TGA_ was observed for CaSCF1919 and CaSCF2828. XRD showed that A’ = Ca samples remained in the perovskite phase with little crystal structure changes before and after TGA. Incongruent Δ*m* was observed upon reduction under low O_2_/Ar in the TGA, potentially due to a reversible phase change, reversible dissociation [28], or induced order/disorder (e.g., in oxygen vacancy) in the material. Post-TGA XRD at room temperature revealed no changes from pre-TGA scans.

#### 3.2.3. TGA Screening 3

**Figure 9:**
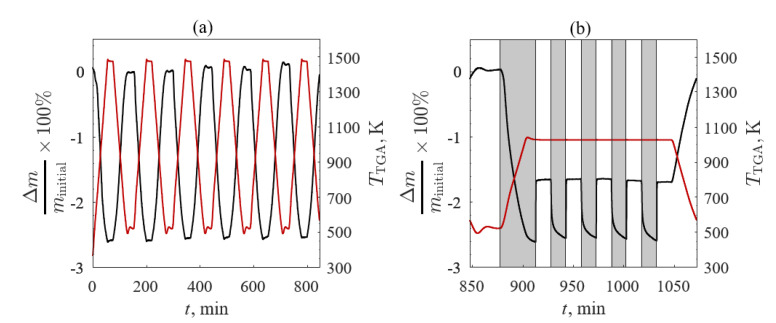
Relative mass change (black line) and temperature (red line) versus time for Ba_0.1_Sr_0.9_Co_0.1_Fe_0.9_O_3−δ_ with five reduction/oxidation cycles with the (**a**) temperature-swing from 523 and 1473 K in 20% O_2_/Ar and (**b**) O_2_ pressure-swing from 20% O_2_/Ar to 100% Ar (gray region) O_2_/Ar at 1023 K for TGA Screening 3.

The Δ*m*/*m*_initial_ (black line) and *T*_TGA_ (red line) versus time for BSCF1919 for TGA Screening 3 are shown in Figure 9 (*m*_initial_ = 107.0 mg) for (a) temperature- and (b) O_2_ pressure-swing cycles. The five temperature-swing cycles between 523 and 1473 K at 20% O_2_/Ar were consistent with TGA Screenings 1 and 2, where the Δ*m*/*m*_initial_ closely followed *T*_TGA_ and equilibrated during the isothermal steps (Figure 9a). During the isothermal O_2_ pressure-swing cycles between 20% O_2_/Ar and 100% Ar (denoted in gray) at 1023 K (Figure 9b), the mass change of the sample was due to Le Chatelier’s Principle. The sample reached equilibrium during oxidation but not during reduction. This was due to the same phenomena observed in TGA Screening 2 where residual O_2_ during the changeover to 100% Ar hindered reduction potential.

The mean Δ*m*/*m*_initial_, mean Δδ and the standard deviation for all the temperature- and O_2_ pressure-swing cycles are presented in Table 4. All A’*_x_*Sr_1−*x*_B’*_y_*Fe_1−*y*_O_3−δ_ had higher redox capacities during the temperature-swings compared to SrFeO_3−δ_ but lower redox capacities compared to SrFeO_3−δ_ during the O_2_ pressure-swings at 1023 K. This was possibly caused by the increased disorder and changes in fugacity associated with A- and/or B-site substitutions compared to SrFeO_3−δ_. The mean Δδ was highest for LSCF1919 during temperature-swings, and all A’*_x_*Sr_1−*x*_B’*_y_*Fe_1−*y*_O_3−δ_ had a lower mean Δδ compared to SrFeO_3−δ_ for the O_2_ pressure-swings. The standard deviations were three orders of magnitude smaller than mean Δδ, indicative of little cycle degradation and a high level of repeatability.

## 4. Discussion

This work outlines a foundational study to examine the redox capacities of SrFeO_3−δ_ with different A- and B-site substitutions. The aim of the study was to identify promising materials to increase redox capacity and stability at reduced temperatures and reaction enthalpies for thermochemical air separation. Systematic analyses extended previous work that examined A’ = La, Ba, Ca and B’ = Co, Cu, Mn. The A-site substitutions assessed the impact of ionic radii and aliovalent substitutions that alter the average B-site oxidation state for site factions of 0 ≤ *x* ≤ 0.2 with A’ = La, Ba, and Ca. B-site substitutions investigated different transitional metal ions capable of increasing redox capacities for B’ = Co, Cr, Cu, and Mn for site fractions of 0 ≤ *y* ≤ 0.2. The stability of promising A- and B-site substitutions over multiple cycles for different O_2_ pressure- and temperature-swings was examined along with changes in morphology with SEM, elemental distributions with EDS, and crystallography and phase identification with XRD. This work provides a strong foundation for developing the solar thermochemical reactors and off-sun oxidation reactors to separate O_2_ from air. A framework for identifying the rate-limiting mechanism(s) for both oxidation and reduction reactions through detailed kinetic analyses was also outlined.

## 5. Conclusions

An A- and B-site substitutional study in SrFeO_3−δ_ (A’*_x_*Sr_1−*x*_B’*_y_*Fe_1−*y*_O_3−δ_) was performed to examine the potential of removing O_2_ from air to produce N_2_ in a two-step solar thermochemical cycle based on reversible reduction/oxidation (redox) reactions. Thermogravimetry was used in three screening studies using temperature- and O_2_ pressures-swings to assess redox capacities and chemical stability. X-ray diffractometry and scanning electron microscopy/energy dispersive spectroscopy were used to characterize particle morphology and elemental dispersion. A’ = Ba and La compositions showed a homogenous distribution of the cations, indicating no phase segregation. A’ = Ba and La compositions showed no evident changes in the particle size, indicating no significant sintering. B’ = Cu, Cr, and Mn substitutions were found to diminish redox capacities compared with SrFeO_3−δ_. La_0.1_Sr_0.9_Co_0.1_Fe_0.9_O_3−δ_ showed the greatest improvement in redox capacities with induced deviation from stoichiometry changes of 0.37 upon reduction, followed by Ba_0.1_Sr_0.9_FeO_3−δ_ and La_0.1_Sr_0.9_FeO_3−δ_ with equal deviation from stoichiometry changes of 0.34. All samples except for A’ = Ca showed continuous and reversible reaction. There is evidence that samples with A’ = Ca underwent reversible phase changes at high temperatures and low O_2_ pressures. Little to no redox capacity degradation was observed during temperature- and pressure-swing cycling for all samples. The high redox capacities, rapid reaction rates, and structural stability are promising initial results for solar thermochemical air separation cycles based on A’*_x_*Sr_1−*x*_B’*_y_*Fe_1−*y*_O_3−δ_ redox reactions.

## Figures and Tables

**Table 1:** Lattice structures from X-ray diffractometry for A- and/or B-site substitutions in SrFeO_3−δ_ (C ≡ cubic, T ≡ tetragonal, O ≡ orthorhombic, M ≡ monoclinic) with secondary phase (s).

Sample	Crystal Structure	PDF-4+ Card #	Secondary Phase (s)
SrFeO_3__−__δ_	T	04-023-5157	N
BSF190	C	00-059-0658	N
BSF280	C	00-059-0658	N
CaSF190	T	04-011-5465	N
CaSF280	O	01-083-3533	N
LSF190	C	04-023-5157	Y
LSF280	C	04-023-5157	Y
SCF019	O	04-011-5466	N
SCF028	T	04-002-0279	N
SCrF019	O	04-020-6506	N
SCrF028	T	04-006-9226	N
SCuF019	T	04-018-8743	N
SCuF028	T	04-021-6591	Y
SMF019	C	04-007-9930	N
SMF028	C	04-006-6180	Y
BSCF1919	C	04-013-0029	Y
BSCF2828	C	04-013-0029	N
CaSCF1919	C	04-008-4374	Y
CaSCF2828	C	04-008-3172	Y
LSCF1919	T	04-002-3182	N
LSCF2828	C	04-016-4840	Y

**Table 2:** Relative mass changes and deviation from stoichiometry changes for A- and/or B-site substitutions in SrFeO_3−δ_ for TGA Screening 1 with temperatures between 573 and 1373 K in 20% O_2_/Ar.

Sample	Δ*m*/*m*_initial_ × 100%	Δδ	Sample	Δ*m*/*m*_initial_ × 100%	Δδ
SrFeO_3-δ_	−2.33	0.28	-	-	-
BSF190	−2.36	0.29	BSF280	−2.23	0.28
LSF190	−2.61	0.32	LSF280	−2.54	0.32
CaSF190	−2.34	0.27	CaSF280	−2.37	0.27
SCF019	−2.31	0.28	SCF028	−2.28	0.28
SCrF019	−1.78	0.21	SCrF028	−1.37	0.16
SCuF019	−1.79	0.22	SCuF028	−1.85	0.23
SMnF019	−1.85	0.22	SMnF028	−1.99	0.24

**Table 3:** Relative mass change and deviation from stoichiometry change for A- and/or B-site substitutions in SrFeO_3−δ_ for TGA Screening 1 with temperatures between 573 and 1373 K under 20% O_2_/Ar and 100% Ar.

Sample	Δ*m*/*m*_initial_ × 100%		*Δ*δ		Sample	Δ*m*/*m*_initial_ × 100%		*Δ*δ	
	20% O_2_/Ar	100% Ar	20% O_2_/Ar	100% Ar		20% O_2_/Ar	100% Ar	20% O_2_/Ar	100% Ar
SrFeO_3-δ_	−2.34	−2.71	0.28	0.32	-	-	-	-	-
BSF190	−2.90	−3.25	0.36	0.40	BSF280	−2.32	−2.57	0.29	0.32
BSCF1919	−2.59	−3.00	0.32	0.37	BSCF2828	−2.33	−2.84	0.29	0.36
LSF190	−2.71	−3.16	0.33	0.39	LSF280	−2.55	−2.96	0.32	0.37
LSCF1919	−2.91	−3.37	0.36	0.42	LSCF2828	−2.72	−3.31	0.34	0.42
CaSF190	−2.50	−2.87	0.29	0.34	CaSF280	−2.53	−2.88	0.29	0.33
CaSCF1919	−2.66	−3.14	0.31	0.37	CaSCF2828	−2.31	−2.94	0.26	0.34
SCF019	−2.21	−2.58	0.27	0.31	SCF028	−2.13	−2.64	0.26	0.32

**Table 4:** Relative mass change and mean and standard deviation of deviation from stoichiometry change for A- and B-site substitutions in SrFeO_3−δ_ for TGA Screening 3 during the temperature- and O_2_ pressure-swings.

Sample	Temperature-Swing			O_2_ Pressure-Swing		
	Δ*m*/*m*_initial_ × 100%	$\bar{\Delta \delta }$	σ_Δδ_	Δ*m*/*m*_initial_ × 100%	$\bar{\Delta \delta }$	σ_Δδ_
SrFeO_3−δ_	−2.60	0.31	0.0015	−1.16	0.14	0.0006
BSF190	−2.75	0.34	0.0035	−1.00	0.12	0.0039
BSCF1919	−2.62	0.33	0.0041	−0.90	0.11	0.0033
LSF190	−2.74	0.34	0.0049	−1.09	0.13	0.0042
LSCF1919	−2.89	0.37	0.0034	−1.03	0.13	0.0045

## Nomenclature

δ	deviation from stoichiometry
σ_Δδ_	standard deviation of the change in deviation from stoichiometry
2θ	X-ray diffraction angle
A	A-site cation
A’	A-site cation substitution
B	B-site cation
B’	B-site cation substitution
C	cubic lattice structure
$Fe_{Fe}^{\times }$	neutral charged Fe ion
$Fe_{Fe}^{\prime }$	negatively charged Fe ion
I	X-ray diffraction peak intensity
M	monoclinic lattice structure
*M*_initial_	stochiometric molar mass of sample
*m*_initial_	initial mass
Δm	change in mass
$M_{O_{2}}$	molar mass of O_2_
O	orthorhombic lattice structure
$O_{O}^{\times }$	neutral charged O ion
T	tetragonal lattice structure
t	time
*T*_TGA_	measured temperature of the TGA
V_Ö_	O^2−^ vacancy
x	A-site substitution fraction
y	B-site substitution fraction
